# (*E*)-4-(2,5-Dimethoxy­benzyl­idene)-2-phenyl-1,3-oxazol-5(4*H*)-one

**DOI:** 10.1107/S1600536809024593

**Published:** 2009-07-04

**Authors:** Abdullah Mohamed Asiri, Seik Weng Ng

**Affiliations:** a Chemistry Department, Faculty of Science, King Abdul Aziz University, Jeddah, Saudi Arabia; b Department of Chemistry, University of Malaya, 50603 Kuala Lumpur, Malaysia

## Abstract

The central aza­lactone ring in the title compound, C_18_H_15_NO_4_, is planar (r.m.s. deviation 0.05, 0.12 Å) in both independent mol­ecules comprising the asymmetric unit. The benzyl­idene substituent is coplanar with this ring [dihedral angle between the planes = 1.8 (1)° in the first mol­ecule and 2.8 (1)° in the second], as is the phenyl substitutent [dihedral angle between rings = 4.6 (1) and 9.7 (1)°, respectively].

## Related literature

For the synthesis of this aza­lactone (which is used in the synthesis of other bioactive compounds), see: Bansal & Jain (1968 ▶); Gulland & Virden (1928 ▶); Hoseini & Jabar (2003 ▶); Khosropour *et al.* (2008 ▶); Neuberger (1948 ▶); Radadia *et al.* (2006 ▶); Solankee *et al.* (2004 ▶); Yakovlev (1950 ▶).
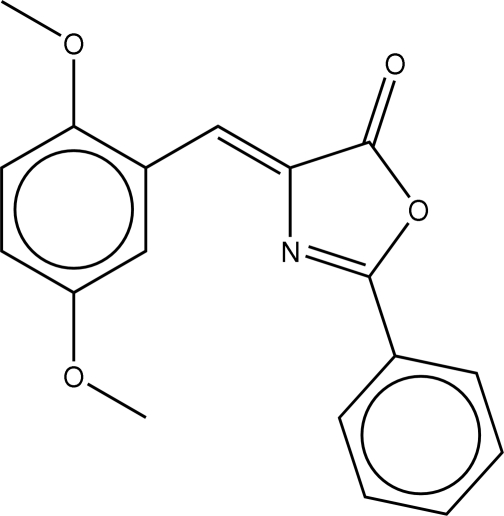


## Experimental

### 

#### Crystal data

C_18_H_15_NO_4_
*M* *_r_* = 309.31
Triclinic, 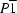

*a* = 7.3893 (2) Å
*b* = 10.6747 (3) Å
*c* = 19.9788 (5) Å
α = 92.408 (2)°
β = 89.780 (2)°
γ = 109.589 (2)°
*V* = 1483.29 (7) Å^3^
*Z* = 4
Mo *K*α radiation
μ = 0.10 mm^−1^
*T* = 140 K
0.35 × 0.25 × 0.05 mm

#### Data collection

Bruker SMART APEX diffractometer
Absorption correction: none
12194 measured reflections
6674 independent reflections
4460 reflections with *I* > 2σ(*I*)
*R*_int_ = 0.029

#### Refinement

*R*[*F* ^2^ > 2σ(*F* ^2^)] = 0.046
*wR*(*F* ^2^) = 0.124
*S* = 1.02
6674 reflections
419 parameters
H-atom parameters constrained
Δρ_max_ = 0.25 e Å^−3^
Δρ_min_ = −0.21 e Å^−3^

### 

Data collection: *APEX2* (Bruker, 2008 ▶); cell refinement: *SAINT* (Bruker, 2008 ▶); data reduction: *SAINT*; program(s) used to solve structure: *SHELXS97* (Sheldrick, 2008 ▶); program(s) used to refine structure: *SHELXL97* (Sheldrick, 2008 ▶); molecular graphics: *X-SEED* (Barbour, 2001 ▶); software used to prepare material for publication: *publCIF* (Westrip, 2009 ▶).

## Supplementary Material

Crystal structure: contains datablocks global, I. DOI: 10.1107/S1600536809024593/tk2485sup1.cif

Structure factors: contains datablocks I. DOI: 10.1107/S1600536809024593/tk2485Isup2.hkl

Additional supplementary materials:  crystallographic information; 3D view; checkCIF report

## Figures and Tables

**Fig. 1.**
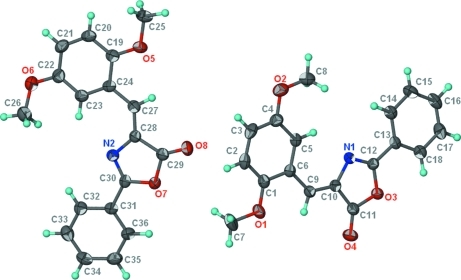
Thermal ellipsoid plot (Barbour, 2001) of the two independent molecules of C18H15NO4 at the 70% probability level; hydrogen atoms are drawn as spheres of arbitrary radius.

## supplementary crystallographic information

### Experimental

Anhydrous sodium acetate (0.23 g, 0.0032 mol) was added to an acetic anhydride (0.12 ml, 0.0028 mol) solution of 2,5-dimethoxybenzaldehyde (1 g, 0.0032 mol) and hippuric acid (0.68 g, 0.0038 mol). The mixture was heated to 353 K for 2 h. The mixture was cooled and ethanol (10 ml) added. The crude azalactone was collected and washed with hot water. Recrystallization from acetone/water (1/1) afforded the pure azalactone as yellow-crystals crystals in 70% yield; m.p. 448 K.

### Refinement

Carbon-bound H-atoms were placed in calculated positions (C—H 0.95–0.98 Å) and were included in the refinement in the riding model approximation with *U*(H) fixed at 1.2–1.5*U*_eq_(C).

### Crystal data

**Table:**

C_18_H_15_NO_4_	*Z* = 4
*M**_r_* = 309.31	*F*(000) = 648
Triclinic, *P*1	*D*_x_ = 1.385 Mg m^−^^3^
Hall symbol: -P 1	Mo *K*α radiation, λ = 0.71073 Å
*a* = 7.3893 (2) Å	Cell parameters from 2608 reflections
*b* = 10.6747 (3) Å	θ = 2.9–27.5°
*c* = 19.9788 (5) Å	µ = 0.10 mm^−^^1^
α = 92.408 (2)°	*T* = 140 K
β = 89.780 (2)°	Plate, yellow-orange
γ = 109.589 (2)°	0.35 × 0.25 × 0.05 mm
*V* = 1483.29 (7) Å^3^	

### Data collection

**Table:**

Bruker SMART APEX diffractometer	4460 reflections with *I* > 2σ(*I*)
Radiation source: fine-focus sealed tube	*R*_int_ = 0.029
graphite	θ_max_ = 27.5°, θ_min_ = 1.0°
ω scans	*h* = −9→9
12194 measured reflections	*k* = −13→13
6674 independent reflections	*l* = −25→24

### Refinement

**Table:**

Refinement on *F*^2^	Primary atom site location: structure-invariant direct methods
Least-squares matrix: full	Secondary atom site location: difference Fourier map
*R*[*F*^2^ > 2σ(*F*^2^)] = 0.046	Hydrogen site location: inferred from neighbouring sites
*wR*(*F*^2^) = 0.124	H-atom parameters constrained
*S* = 1.02	*w* = 1/[σ^2^(*F*_o_^2^) + (0.0549*P*)^2^ + 0.1872*P*] where *P* = (*F*_o_^2^ + 2*F*_c_^2^)/3
6674 reflections	(Δ/σ)_max_ = 0.001
419 parameters	Δρ_max_ = 0.25 e Å^−^^3^
0 restraints	Δρ_min_ = −0.21 e Å^−^^3^

### Fractional atomic coordinates and isotropic or equivalent isotropic displacement parameters (Å^2^)

**Table:**

	*x*	*y*	*z*	*U*_iso_*/*U*_eq_	
O1	0.33728 (19)	0.62721 (13)	0.40379 (6)	0.0310 (3)	
O2	0.6638 (2)	1.10751 (14)	0.28431 (7)	0.0382 (4)	
O3	0.86578 (18)	1.00629 (12)	0.62162 (6)	0.0263 (3)	
O4	0.6850 (2)	0.78813 (14)	0.62357 (7)	0.0377 (4)	
O5	0.3182 (2)	0.87366 (13)	−0.04754 (6)	0.0305 (3)	
O6	0.4181 (2)	0.43230 (14)	−0.17481 (7)	0.0365 (4)	
O7	0.10590 (18)	0.44231 (12)	0.16427 (6)	0.0256 (3)	
O8	0.1178 (2)	0.65709 (13)	0.16969 (6)	0.0333 (3)	
N1	0.8113 (2)	1.05526 (14)	0.51618 (7)	0.0226 (3)	
N2	0.1863 (2)	0.41791 (15)	0.05614 (7)	0.0231 (3)	
C1	0.4144 (3)	0.74516 (19)	0.37318 (9)	0.0254 (4)	
C2	0.3736 (3)	0.7636 (2)	0.30708 (9)	0.0293 (4)	
H2	0.2871	0.6922	0.2810	0.035*	
C3	0.4585 (3)	0.8855 (2)	0.27937 (9)	0.0319 (5)	
H3	0.4295	0.8974	0.2343	0.038*	
C4	0.5857 (3)	0.99073 (19)	0.31638 (9)	0.0286 (4)	
C5	0.6260 (3)	0.97409 (19)	0.38221 (9)	0.0247 (4)	
H5	0.7120	1.0466	0.4078	0.030*	
C6	0.5414 (3)	0.85146 (18)	0.41178 (9)	0.0223 (4)	
C7	0.2236 (3)	0.5148 (2)	0.36339 (11)	0.0389 (5)	
H7A	0.1896	0.4348	0.3896	0.058*	
H7B	0.2971	0.5031	0.3241	0.058*	
H7C	0.1061	0.5294	0.3487	0.058*	
C8	0.7886 (3)	1.2170 (2)	0.32279 (10)	0.0376 (5)	
H8A	0.8295	1.2958	0.2956	0.056*	
H8B	0.9015	1.1959	0.3370	0.056*	
H8C	0.7209	1.2349	0.3624	0.056*	
C9	0.5850 (3)	0.83215 (18)	0.48039 (9)	0.0229 (4)	
H9	0.5230	0.7457	0.4963	0.028*	
C10	0.7022 (3)	0.92109 (18)	0.52466 (9)	0.0227 (4)	
C11	0.7383 (3)	0.88791 (19)	0.59291 (9)	0.0257 (4)	
C12	0.8990 (3)	1.09834 (18)	0.57233 (8)	0.0218 (4)	
C13	1.0307 (3)	1.23148 (18)	0.58871 (9)	0.0230 (4)	
C14	1.0778 (3)	1.32452 (19)	0.53919 (10)	0.0302 (4)	
H14	1.0184	1.3021	0.4962	0.036*	
C15	1.2108 (3)	1.4497 (2)	0.55242 (11)	0.0357 (5)	
H15	1.2436	1.5130	0.5185	0.043*	
C16	1.2964 (3)	1.4825 (2)	0.61529 (11)	0.0346 (5)	
H16	1.3898	1.5678	0.6241	0.042*	
C17	1.2466 (3)	1.3922 (2)	0.66500 (10)	0.0330 (5)	
H17	1.3029	1.4164	0.7084	0.040*	
C18	1.1146 (3)	1.26620 (19)	0.65212 (9)	0.0279 (4)	
H18	1.0815	1.2037	0.6864	0.033*	
C19	0.3386 (3)	0.76448 (19)	−0.08112 (9)	0.0257 (4)	
C20	0.3947 (3)	0.7645 (2)	−0.14757 (9)	0.0291 (4)	
H20	0.4177	0.8421	−0.1725	0.035*	
C21	0.4172 (3)	0.6517 (2)	−0.17748 (9)	0.0306 (5)	
H21	0.4555	0.6521	−0.2229	0.037*	
C22	0.3841 (3)	0.5380 (2)	−0.14169 (9)	0.0281 (4)	
C23	0.3232 (3)	0.53509 (19)	−0.07638 (9)	0.0257 (4)	
H23	0.2973	0.4559	−0.0526	0.031*	
C24	0.2991 (3)	0.64866 (18)	−0.04463 (9)	0.0235 (4)	
C25	0.3610 (3)	0.99354 (19)	−0.08297 (10)	0.0335 (5)	
H25A	0.3484	1.0648	−0.0527	0.050*	
H25B	0.2713	0.9792	−0.1208	0.050*	
H25C	0.4928	1.0189	−0.0996	0.050*	
C26	0.3406 (3)	0.3058 (2)	−0.14667 (11)	0.0375 (5)	
H26A	0.3596	0.2376	−0.1776	0.056*	
H26B	0.2030	0.2861	−0.1390	0.056*	
H26C	0.4059	0.3063	−0.1040	0.056*	
C27	0.2365 (3)	0.64931 (19)	0.02420 (9)	0.0250 (4)	
H27	0.2280	0.7313	0.0416	0.030*	
C28	0.1887 (3)	0.54878 (18)	0.06689 (9)	0.0229 (4)	
C29	0.1353 (3)	0.56526 (19)	0.13700 (9)	0.0255 (4)	
C30	0.1396 (3)	0.36291 (18)	0.11257 (8)	0.0224 (4)	
C31	0.1247 (3)	0.22752 (18)	0.12781 (9)	0.0235 (4)	
C32	0.1394 (3)	0.14044 (19)	0.07566 (9)	0.0272 (4)	
H32	0.1534	0.1684	0.0308	0.033*	
C33	0.1334 (3)	0.0136 (2)	0.08943 (10)	0.0312 (5)	
H33	0.1422	−0.0464	0.0541	0.037*	
C34	0.1146 (3)	−0.0264 (2)	0.15501 (10)	0.0325 (5)	
H34	0.1108	−0.1137	0.1643	0.039*	
C35	0.1013 (3)	0.0595 (2)	0.20680 (10)	0.0340 (5)	
H35	0.0894	0.0316	0.2516	0.041*	
C36	0.1054 (3)	0.1865 (2)	0.19328 (10)	0.0301 (4)	
H36B	0.0950	0.2456	0.2288	0.036*	

### Atomic displacement parameters (Å^2^)

**Table:**

	*U*^11^	*U*^22^	*U*^33^	*U*^12^	*U*^13^	*U*^23^
O1	0.0311 (8)	0.0234 (7)	0.0329 (7)	0.0022 (6)	−0.0059 (6)	−0.0049 (6)
O2	0.0464 (9)	0.0380 (9)	0.0277 (7)	0.0101 (7)	−0.0024 (6)	0.0087 (6)
O3	0.0310 (7)	0.0217 (7)	0.0221 (6)	0.0032 (6)	−0.0041 (5)	0.0010 (5)
O4	0.0487 (9)	0.0254 (8)	0.0309 (8)	0.0012 (7)	−0.0068 (7)	0.0067 (6)
O5	0.0376 (8)	0.0238 (7)	0.0299 (7)	0.0094 (6)	0.0004 (6)	0.0072 (6)
O6	0.0440 (9)	0.0334 (8)	0.0300 (8)	0.0108 (7)	0.0050 (6)	−0.0014 (6)
O7	0.0309 (7)	0.0246 (7)	0.0213 (6)	0.0094 (6)	−0.0002 (5)	0.0011 (5)
O8	0.0471 (9)	0.0265 (8)	0.0269 (7)	0.0135 (7)	−0.0043 (6)	−0.0043 (6)
N1	0.0245 (8)	0.0190 (8)	0.0235 (8)	0.0061 (7)	0.0005 (6)	−0.0001 (6)
N2	0.0233 (8)	0.0229 (8)	0.0229 (8)	0.0072 (7)	−0.0025 (6)	0.0021 (6)
C1	0.0245 (10)	0.0255 (10)	0.0271 (10)	0.0099 (9)	0.0007 (8)	−0.0029 (8)
C2	0.0278 (10)	0.0351 (12)	0.0251 (10)	0.0120 (9)	−0.0052 (8)	−0.0089 (8)
C3	0.0358 (12)	0.0434 (13)	0.0196 (9)	0.0178 (10)	−0.0037 (8)	−0.0014 (9)
C4	0.0335 (11)	0.0301 (11)	0.0252 (10)	0.0144 (9)	0.0013 (8)	0.0039 (8)
C5	0.0248 (10)	0.0258 (10)	0.0234 (9)	0.0088 (8)	−0.0012 (7)	−0.0026 (8)
C6	0.0208 (9)	0.0247 (10)	0.0226 (9)	0.0096 (8)	0.0009 (7)	−0.0010 (7)
C7	0.0361 (12)	0.0265 (11)	0.0477 (13)	0.0036 (10)	−0.0088 (10)	−0.0112 (10)
C8	0.0441 (13)	0.0316 (12)	0.0355 (12)	0.0099 (11)	0.0034 (10)	0.0078 (9)
C9	0.0232 (10)	0.0203 (10)	0.0248 (9)	0.0067 (8)	0.0022 (7)	−0.0001 (7)
C10	0.0241 (10)	0.0216 (10)	0.0228 (9)	0.0083 (8)	0.0016 (7)	−0.0001 (7)
C11	0.0280 (10)	0.0224 (10)	0.0249 (9)	0.0061 (9)	−0.0032 (8)	−0.0004 (8)
C12	0.0228 (9)	0.0223 (10)	0.0211 (9)	0.0088 (8)	0.0018 (7)	0.0012 (7)
C13	0.0207 (9)	0.0212 (10)	0.0264 (9)	0.0063 (8)	−0.0003 (7)	−0.0031 (7)
C14	0.0334 (11)	0.0255 (11)	0.0291 (10)	0.0066 (9)	−0.0017 (8)	−0.0012 (8)
C15	0.0388 (12)	0.0239 (11)	0.0402 (12)	0.0045 (10)	0.0039 (9)	0.0040 (9)
C16	0.0290 (11)	0.0227 (11)	0.0485 (13)	0.0049 (9)	−0.0021 (9)	−0.0068 (9)
C17	0.0305 (11)	0.0292 (11)	0.0373 (11)	0.0087 (9)	−0.0109 (9)	−0.0088 (9)
C18	0.0281 (11)	0.0249 (10)	0.0302 (10)	0.0086 (9)	−0.0028 (8)	−0.0009 (8)
C19	0.0240 (10)	0.0255 (10)	0.0259 (10)	0.0059 (8)	−0.0061 (8)	0.0032 (8)
C20	0.0271 (10)	0.0282 (11)	0.0268 (10)	0.0017 (9)	−0.0048 (8)	0.0077 (8)
C21	0.0268 (11)	0.0377 (12)	0.0211 (10)	0.0022 (9)	−0.0015 (8)	0.0029 (8)
C22	0.0243 (10)	0.0315 (11)	0.0256 (10)	0.0056 (9)	−0.0036 (8)	−0.0021 (8)
C23	0.0226 (10)	0.0253 (10)	0.0261 (10)	0.0035 (8)	−0.0030 (7)	0.0032 (8)
C24	0.0192 (9)	0.0251 (10)	0.0242 (9)	0.0043 (8)	−0.0035 (7)	0.0039 (8)
C25	0.0352 (12)	0.0254 (11)	0.0399 (12)	0.0088 (9)	−0.0002 (9)	0.0107 (9)
C26	0.0383 (12)	0.0307 (12)	0.0417 (12)	0.0098 (10)	0.0010 (10)	−0.0030 (9)
C27	0.0267 (10)	0.0231 (10)	0.0252 (9)	0.0084 (8)	−0.0057 (8)	0.0011 (8)
C28	0.0236 (10)	0.0234 (10)	0.0210 (9)	0.0071 (8)	−0.0032 (7)	0.0000 (7)
C29	0.0262 (10)	0.0236 (10)	0.0245 (9)	0.0053 (8)	−0.0052 (7)	0.0011 (8)
C30	0.0204 (9)	0.0258 (10)	0.0214 (9)	0.0085 (8)	−0.0024 (7)	−0.0014 (7)
C31	0.0202 (9)	0.0236 (10)	0.0263 (9)	0.0066 (8)	−0.0011 (7)	0.0034 (8)
C32	0.0270 (10)	0.0290 (11)	0.0264 (10)	0.0102 (9)	−0.0018 (8)	0.0012 (8)
C33	0.0288 (11)	0.0288 (11)	0.0367 (11)	0.0111 (9)	−0.0057 (8)	−0.0045 (9)
C34	0.0303 (11)	0.0240 (11)	0.0424 (12)	0.0075 (9)	−0.0049 (9)	0.0065 (9)
C35	0.0385 (12)	0.0321 (12)	0.0299 (11)	0.0088 (10)	0.0007 (9)	0.0098 (9)
C36	0.0339 (11)	0.0284 (11)	0.0271 (10)	0.0090 (9)	0.0041 (8)	0.0049 (8)

### Geometric parameters (Å, °)

**Table:**

O1—C1	1.366 (2)	C14—C15	1.383 (3)
O1—C7	1.430 (2)	C14—H14	0.9500
O2—C4	1.371 (2)	C15—C16	1.386 (3)
O2—C8	1.419 (2)	C15—H15	0.9500
O3—C12	1.383 (2)	C16—C17	1.375 (3)
O3—C11	1.399 (2)	C16—H16	0.9500
O4—C11	1.199 (2)	C17—C18	1.386 (3)
O5—C19	1.372 (2)	C17—H17	0.9500
O5—C25	1.429 (2)	C18—H18	0.9500
O6—C22	1.378 (2)	C19—C20	1.389 (3)
O6—C26	1.418 (2)	C19—C24	1.406 (3)
O7—C30	1.383 (2)	C20—C21	1.381 (3)
O7—C29	1.391 (2)	C20—H20	0.9500
O8—C29	1.198 (2)	C21—C22	1.384 (3)
N1—C12	1.286 (2)	C21—H21	0.9500
N1—C10	1.405 (2)	C22—C23	1.377 (3)
N2—C30	1.285 (2)	C23—C24	1.408 (3)
N2—C28	1.399 (2)	C23—H23	0.9500
C1—C2	1.392 (3)	C24—C27	1.450 (2)
C1—C6	1.408 (2)	C25—H25A	0.9800
C2—C3	1.380 (3)	C25—H25B	0.9800
C2—H2	0.9500	C25—H25C	0.9800
C3—C4	1.385 (3)	C26—H26A	0.9800
C3—H3	0.9500	C26—H26B	0.9800
C4—C5	1.382 (2)	C26—H26C	0.9800
C5—C6	1.402 (3)	C27—C28	1.351 (2)
C5—H5	0.9500	C27—H27	0.9500
C6—C9	1.448 (2)	C28—C29	1.473 (2)
C7—H7A	0.9800	C30—C31	1.457 (2)
C7—H7B	0.9800	C31—C36	1.389 (3)
C7—H7C	0.9800	C31—C32	1.395 (2)
C8—H8A	0.9800	C32—C33	1.380 (3)
C8—H8B	0.9800	C32—H32	0.9500
C8—H8C	0.9800	C33—C34	1.387 (3)
C9—C10	1.348 (2)	C33—H33	0.9500
C9—H9	0.9500	C34—C35	1.379 (3)
C10—C11	1.471 (2)	C34—H34	0.9500
C12—C13	1.453 (3)	C35—C36	1.384 (3)
C13—C14	1.391 (3)	C35—H35	0.9500
C13—C18	1.393 (2)	C36—H36B	0.9500
			
C1—O1—C7	117.44 (15)	C18—C17—H17	119.9
C4—O2—C8	116.75 (15)	C17—C18—C13	119.78 (18)
C12—O3—C11	105.55 (13)	C17—C18—H18	120.1
C19—O5—C25	117.65 (15)	C13—C18—H18	120.1
C22—O6—C26	117.31 (15)	O5—C19—C20	123.37 (17)
C30—O7—C29	105.28 (13)	O5—C19—C24	116.41 (16)
C12—N1—C10	105.88 (15)	C20—C19—C24	120.22 (17)
C30—N2—C28	105.50 (14)	C21—C20—C19	120.04 (18)
O1—C1—C2	123.37 (17)	C21—C20—H20	120.0
O1—C1—C6	116.80 (16)	C19—C20—H20	120.0
C2—C1—C6	119.82 (18)	C20—C21—C22	120.46 (18)
C3—C2—C1	120.05 (18)	C20—C21—H21	119.8
C3—C2—H2	120.0	C22—C21—H21	119.8
C1—C2—H2	120.0	O6—C22—C23	123.41 (18)
C2—C3—C4	120.91 (18)	O6—C22—C21	116.33 (17)
C2—C3—H3	119.5	C23—C22—C21	120.26 (18)
C4—C3—H3	119.5	C22—C23—C24	120.47 (18)
O2—C4—C5	123.80 (18)	C22—C23—H23	119.8
O2—C4—C3	116.59 (17)	C24—C23—H23	119.8
C5—C4—C3	119.61 (19)	C19—C24—C23	118.50 (17)
C4—C5—C6	120.76 (17)	C19—C24—C27	119.58 (16)
C4—C5—H5	119.6	C23—C24—C27	121.92 (17)
C6—C5—H5	119.6	O5—C25—H25A	109.5
C5—C6—C1	118.84 (16)	O5—C25—H25B	109.5
C5—C6—C9	121.28 (16)	H25A—C25—H25B	109.5
C1—C6—C9	119.87 (17)	O5—C25—H25C	109.5
O1—C7—H7A	109.5	H25A—C25—H25C	109.5
O1—C7—H7B	109.5	H25B—C25—H25C	109.5
H7A—C7—H7B	109.5	O6—C26—H26A	109.5
O1—C7—H7C	109.5	O6—C26—H26B	109.5
H7A—C7—H7C	109.5	H26A—C26—H26B	109.5
H7B—C7—H7C	109.5	O6—C26—H26C	109.5
O2—C8—H8A	109.5	H26A—C26—H26C	109.5
O2—C8—H8B	109.5	H26B—C26—H26C	109.5
H8A—C8—H8B	109.5	C28—C27—C24	128.61 (17)
O2—C8—H8C	109.5	C28—C27—H27	115.7
H8A—C8—H8C	109.5	C24—C27—H27	115.7
H8B—C8—H8C	109.5	C27—C28—N2	129.08 (16)
C10—C9—C6	128.36 (18)	C27—C28—C29	122.81 (16)
C10—C9—H9	115.8	N2—C28—C29	108.07 (15)
C6—C9—H9	115.8	O8—C29—O7	121.70 (17)
C9—C10—N1	128.96 (17)	O8—C29—C28	133.35 (18)
C9—C10—C11	123.11 (17)	O7—C29—C28	104.95 (15)
N1—C10—C11	107.92 (15)	N2—C30—O7	116.19 (16)
O4—C11—O3	121.15 (16)	N2—C30—C31	126.59 (16)
O4—C11—C10	134.02 (18)	O7—C30—C31	117.19 (15)
O3—C11—C10	104.83 (15)	C36—C31—C32	119.94 (17)
N1—C12—O3	115.80 (16)	C36—C31—C30	121.12 (17)
N1—C12—C13	127.03 (16)	C32—C31—C30	118.87 (16)
O3—C12—C13	117.16 (15)	C33—C32—C31	119.74 (18)
C14—C13—C18	119.62 (17)	C33—C32—H32	120.1
C14—C13—C12	118.97 (16)	C31—C32—H32	120.1
C18—C13—C12	121.38 (17)	C32—C33—C34	119.89 (19)
C15—C14—C13	120.12 (18)	C32—C33—H33	120.1
C15—C14—H14	119.9	C34—C33—H33	120.1
C13—C14—H14	119.9	C35—C34—C33	120.63 (19)
C14—C15—C16	119.8 (2)	C35—C34—H34	119.7
C14—C15—H15	120.1	C33—C34—H34	119.7
C16—C15—H15	120.1	C34—C35—C36	119.79 (19)
C17—C16—C15	120.32 (19)	C34—C35—H35	120.1
C17—C16—H16	119.8	C36—C35—H35	120.1
C15—C16—H16	119.8	C35—C36—C31	120.01 (18)
C16—C17—C18	120.28 (18)	C35—C36—H36B	120.0
C16—C17—H17	119.9	C31—C36—H36B	120.0
			
C7—O1—C1—C2	6.1 (3)	C25—O5—C19—C20	−1.3 (3)
C7—O1—C1—C6	−173.82 (16)	C25—O5—C19—C24	178.80 (16)
O1—C1—C2—C3	−179.40 (17)	O5—C19—C20—C21	178.28 (17)
C6—C1—C2—C3	0.5 (3)	C24—C19—C20—C21	−1.8 (3)
C1—C2—C3—C4	0.3 (3)	C19—C20—C21—C22	0.0 (3)
C8—O2—C4—C5	−1.9 (3)	C26—O6—C22—C23	16.6 (3)
C8—O2—C4—C3	177.74 (17)	C26—O6—C22—C21	−164.31 (17)
C2—C3—C4—O2	179.50 (16)	C20—C21—C22—O6	−177.32 (17)
C2—C3—C4—C5	−0.9 (3)	C20—C21—C22—C23	1.8 (3)
O2—C4—C5—C6	−179.69 (17)	O6—C22—C23—C24	177.30 (17)
C3—C4—C5—C6	0.7 (3)	C21—C22—C23—C24	−1.8 (3)
C4—C5—C6—C1	0.0 (3)	O5—C19—C24—C23	−178.26 (16)
C4—C5—C6—C9	178.98 (17)	C20—C19—C24—C23	1.9 (3)
O1—C1—C6—C5	179.25 (15)	O5—C19—C24—C27	1.3 (2)
C2—C1—C6—C5	−0.6 (3)	C20—C19—C24—C27	−178.60 (16)
O1—C1—C6—C9	0.3 (2)	C22—C23—C24—C19	0.0 (3)
C2—C1—C6—C9	−179.61 (17)	C22—C23—C24—C27	−179.57 (17)
C5—C6—C9—C10	0.9 (3)	C19—C24—C27—C28	178.73 (18)
C1—C6—C9—C10	179.88 (18)	C23—C24—C27—C28	−1.7 (3)
C6—C9—C10—N1	0.4 (3)	C24—C27—C28—N2	−0.2 (3)
C6—C9—C10—C11	−178.41 (17)	C24—C27—C28—C29	177.19 (17)
C12—N1—C10—C9	179.93 (18)	C30—N2—C28—C27	177.07 (19)
C12—N1—C10—C11	−1.09 (19)	C30—N2—C28—C29	−0.60 (19)
C12—O3—C11—O4	178.61 (18)	C30—O7—C29—O8	−179.66 (17)
C12—O3—C11—C10	−0.44 (18)	C30—O7—C29—C28	−0.32 (17)
C9—C10—C11—O4	1.1 (3)	C27—C28—C29—O8	2.0 (3)
N1—C10—C11—O4	−177.9 (2)	N2—C28—C29—O8	179.8 (2)
C9—C10—C11—O3	180.00 (16)	C27—C28—C29—O7	−177.27 (16)
N1—C10—C11—O3	0.94 (19)	N2—C28—C29—O7	0.58 (18)
C10—N1—C12—O3	0.9 (2)	C28—N2—C30—O7	0.4 (2)
C10—N1—C12—C13	179.58 (16)	C28—N2—C30—C31	−177.57 (17)
C11—O3—C12—N1	−0.3 (2)	C29—O7—C30—N2	−0.1 (2)
C11—O3—C12—C13	−179.11 (15)	C29—O7—C30—C31	178.13 (15)
N1—C12—C13—C14	−2.9 (3)	N2—C30—C31—C36	168.82 (18)
O3—C12—C13—C14	175.82 (16)	O7—C30—C31—C36	−9.2 (3)
N1—C12—C13—C18	179.07 (18)	N2—C30—C31—C32	−8.1 (3)
O3—C12—C13—C18	−2.2 (2)	O7—C30—C31—C32	173.89 (15)
C18—C13—C14—C15	1.6 (3)	C36—C31—C32—C33	0.4 (3)
C12—C13—C14—C15	−176.45 (17)	C30—C31—C32—C33	177.37 (17)
C13—C14—C15—C16	−0.4 (3)	C31—C32—C33—C34	−0.6 (3)
C14—C15—C16—C17	−1.4 (3)	C32—C33—C34—C35	0.1 (3)
C15—C16—C17—C18	1.9 (3)	C33—C34—C35—C36	0.5 (3)
C16—C17—C18—C13	−0.7 (3)	C34—C35—C36—C31	−0.6 (3)
C14—C13—C18—C17	−1.1 (3)	C32—C31—C36—C35	0.2 (3)
C12—C13—C18—C17	176.95 (17)	C30—C31—C36—C35	−176.72 (18)

## References

[bb1] Bansal, R. K. & Jain, J. K. (1968). *Synthesis*, pp. 840–842.

[bb2] Barbour, L. J. (2001). *J. Supramol. Chem.* **1**, 189–191.

[bb3] Bruker (2008). *APEX2* and *SAINT* Bruker AXS Inc., Madison, Wisconsin, USA.

[bb4] Gulland, J. M. & Virden, C. J. (1928). *J. Chem. Soc.* pp. 478–486.

[bb5] Hoseini, J. & Jabar, S. (2003). *J. Chem. Res. (S)*, pp. 638–641.

[bb6] Khosropour, A. R., Khodaei, M. M. & Jomor, S. J. H. (2008). *J. Heterocycl. Chem.* **45**, 683–686.

[bb7] Neuberger, A. (1948). *Biochem. J.* **43**, 599–605. 10.1042/bj0430599 PMC1274782 16748458

[bb8] Radadia, V. R., Purohit, D. M. & Patolia, V. N. (2006). *J. Inst. Chem. (India)*, **78**, 14–16.

[bb9] Sheldrick, G. M. (2008). *Acta Cryst.* A**64**, 112–122. 10.1107/S0108767307043930 18156677

[bb10] Solankee, A., Kapadia, K., Thakor, I. & Patel, J. (2004). *Asian J. Chem.* **16**, 917–920.

[bb11] Westrip, S. P. (2009). *publCIF* In preparation.

[bb12] Yakovlev, V. G. (1950). *Zh. Obshch. Khim.* **20**, 361–367.

